# Exosomal noncoding RNAs in gynecological cancers: implications for therapy resistance and biomarkers

**DOI:** 10.3389/fonc.2024.1349474

**Published:** 2024-04-26

**Authors:** Changfen Xu, Peiyao Xu, Jiaqi Zhang, Sheng He, Tingting Hua, Aiwu Huang

**Affiliations:** Department of Gynecology and Obstetrics , Hangzhou Lin'an Traditional Chinese Medicine Hospital, Hangzhou, Zhejiang, China

**Keywords:** exosomes, ncRNAs, gynecologic tumors, targeted therapy, biomarkers

## Abstract

Gynecologic cancers, including ovarian cancer (OC), cervical cancer (CC), and endometrial cancer (EC), pose a serious threat to women’s health and quality of life due to their high incidence and lethality. Therapeutic resistance in tumors refers to reduced sensitivity of tumor cells to therapeutic drugs or radiation, which compromises the efficacy of treatment or renders it ineffective. Therapeutic resistance significantly contributes to treatment failure in gynecologic tumors, although the specific molecular mechanisms remain unclear. Exosomes are nanoscale vesicles released and received by distinct kinds of cells. Exosomes contain proteins, lipids, and RNAs closely linked to their origins and functions. Recent studies have demonstrated that exosomal ncRNAs may be involved in intercellular communication and can modulate the progression of tumorigenesis, aggravation and metastasis, tumor microenvironment (TME), and drug resistance. Besides, exosomal ncRNAs also have the potential to become significant diagnostic and prognostic biomarkers in various of diseases. In this paper, we reviewed the biological roles and mechanisms of exosomal ncRNAs in the drug resistance of gynecologic tumors, as well as explored the potential of exosomal ncRNAs acting as the liquid biopsy molecular markers in gynecologic cancers.

## 1 Introduction

Cervical cancer (CC) is one of the most common malignant cancer in the female reproductive system, which have seriously jeopardized womenkind’s physical and mental health recently (1, 2). CC is mainly caused by human papillomavirus infection (3, 4) and its morbidity and mortality have not declined markedly (5–8).Treatment options for early and locally invasive CC involve radical hysterectomy, radical hysterectomy with pelvic lymph node dissection, synchronized chemotherapy and radiotherapy, and systemic therapy for distant metastatic CC (9–11). Endometrial cancer (EC), occurring in the endometrium, is a primary epithelial malignant tumor. Risk ingredient for EC consist of estrogen-secreting ovarian tumors, ovarian dysfunction, metabolic diseases, advanced age, early sexual life, infertility, delayed menopause, and carrying EC-related susceptibility genes (12, 13). Nowadays, the procedure of EC is chiefly based on surgery and radiotherapy, since targeted procedure and immunotherapy have been by degrees applied in the clinical first-line therapy (14). Ovarian cancer (OC) is one of the most common malignancies in women and the deadliest gynecologic cancer (15, 16). Recently, the incidence of OC shows a dramatic increase globally because of population growth, increased cancer risk factors, and declined pregnancy and lactation (17). OC symptoms are frequently poorly characterized and there is a lack of definitive screening tools, over 70% of OCs are diagnosed at the stage III or IV (18–20). The main therapy for OC remains surgery relative to platinum-based chemotherapy (21–24). While metastatic OC are not easy or impossible to cut cleanly become residual cancer foci, which are the root reason of future recurrence and drug resistance (25, 26). Overall, the resistance of radiotherapy or chemotherapy in gynecologic tumors significantly contribute to the tumor recurrence and metastasis and the efficacy of treatment (27). Hence, exploring the molecular mechanism of the therapeutic resistance of gynecologic tumors is urgent.

Exosomes are a subset of extracellular vesicles (EVs) that contain proteins, nucleic acids, metabolites and other substance and serve as an essential role in cellular communication (28–31). Exosomes are crucial in many physiological processes including tissue repair, immune response, stem cell maintenance, pathological processes in the central nervous system and cardiovascular diseases, and inflammatory responses (32–39). Additionally, exosomes also act as vital roles between tumor cells and stromal cells by delivering content associated with genetic information in tumor microenvironment (TME) (40, 41). Exosomes’ biocompatibility and bilayer lipid structure decrease immunogenicity and protect against genetic degradation, making them attractive as therapeutic vectors (42, 43). Noncoding RNAs (ncRNAs) are a class of RNA molecules with minimal protein-coding ability but important regulatory functions. NcRNAs mainly contain microRNAs (miRNAs), long noncoding RNAs (lncRNAs), and circular RNAs (circRNAs). MiRNAs are a unique class of small ncRNAs that can modulate the regulation of gene expression at post-transcriptional level (44). LncRNAs are a class of RNAs longer than 200 nucleotides and closely associated with the initiation and progression of diseases (45, 46). CircRNAs are a newly ncRNAs and exert a great stability due to their closed-loop structures and exhibit cell- or tissue-specific features (47). CircRNAs are abnormal expressed in various cancers and can modulate the progression of tumorigenesis and metastasis and may be the novel biomarkers and therapeutic targets. Recent studies have implied that exosomal ncRNAs exert essential roles in the initiation and development of cancers including gynecologic cancers. Exosomal circWHSC1 could be transferred from OC cells to peritoneal mesothelial cells and promotes peritoneal dissemination by sponging miR-145/miR-1182 and up-regulating the expression of MUC1 and hTERT (48). Plasma exosomal miR-30d-5p and let-7d-3p are valuable diagnostic biomarkers for distinguishing patients in cervical intraepithelial neoplasia (CIN) II+ group from those in CIN I- group and might be the non-invasive screening of CC (49). Besides, exosomal ncRNAscan also regulate the therapeutic resistance of cancers (50–52).

Overall, we aimed to review the biological roles and mechanisms of exosomal ncRNAs in therapeutic resistance, diagnosis and prognostic assessment of gynecologic tumors, which might provide a new theoretical basis for the clinical diagnosis and therapy of gynecologic cancers.

## 2 The overview of ncRNAs

### 2.1 MiRNAs

**Figure 1:**
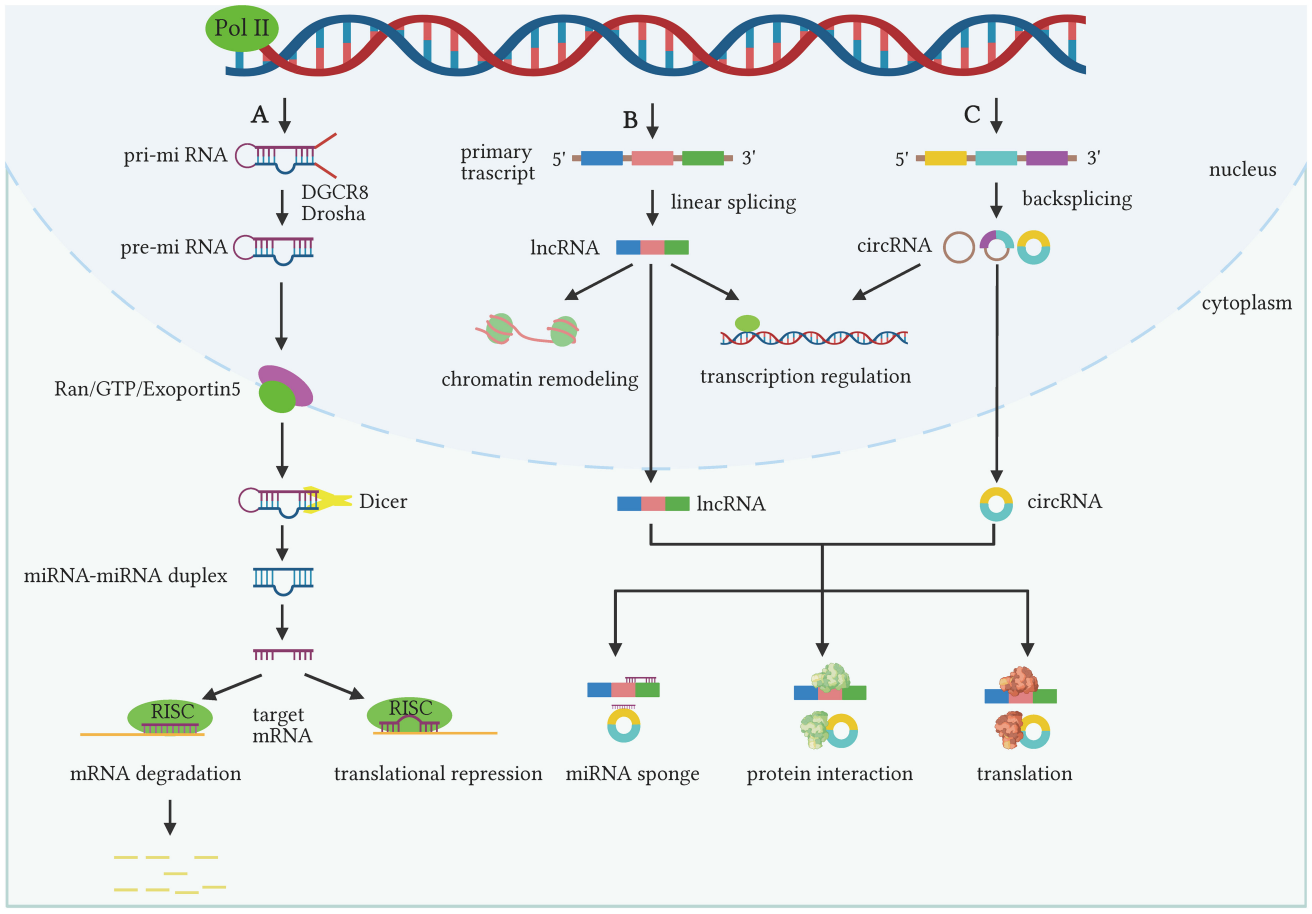
The biogenesis and biological function of miRNAs, lncRNAs and circRNAs. **(A)** The genes for miRNAs are transcribed by polymerase II into primary miRNAs (pri-miRNAs), which can be cleaved by the DGCR8 and Drosha complexes to form precursor miRNAs (pre-miRNAs). The Ran/GTP/Exportin-5 complex can transport pre-miRNA from the nucleus to the cytoplasm and forms miRNA-miRNA double-stranded bodies by Dicer treatment. The mature miRNA is loaded into the RNA-induced silencing complex (RISC) protein complex and directly target mRNA to induce the degradation or translational repression of mRNA. **(B, C)** LncRNAs and circRNAs can be generated from primary transcripts by linear splicing and reverse splicing, respectively. In the nucleus, they can affect the expression of key genes by remodeling chromatin and regulating gene transcription. In the cytoplasm, they can exert biological roles via serving as miRNA sponges, interacting with proteins or encoding peptides. (The figure was generated by Biorender).

MiRNAs are endogenous noncoding single-stranded RNAs located in introns and 19-28 nucleotides in length (53, 54). The process of miRNA generation involves the transcription of miRNAs in the nucleus into a long transcript called primary miRNA (pri-miRNA). The pri-miRNA is subsequently cleaved by the Drosha RNase III nucleic acid endonuclease, forming an intermediate miRNA called precursor miRNA (pre-miRNA). Then the pre-miRNA can be exported to the cytoplasm, where it undergoes a second cleavage by the Dicer ribonuclease to mature into functional miRNAs. The pre-miRNAs are about 22 nucleotides long and can modulate the translation process or degradation of mRNAs by binding to the 3’ untranslated region (3’ UTR). MiRNAs are involved in various of biological processes such as growth, differentiation, and motility (55–57). Moreover, a single miRNA can target multiple distinct genes. While the same gene may be targeted by a group of miRNAs due to they can be only exchanged between cells do not need to be fully complementary in recognizing target genes (58, 59). The biogenesis of miRNAs was displayed in **Figure 1**.

### 2.2 LncRNAs

LncRNAs are a string of ncRNAs greater than 200 bp in length and have little ability to encode proteins. Similar to protein-coding mRNAs, lncRNAs are transcribed by RNA polymerase II and undergo post-transcriptional modifications like 5’ capping polyadenylation and splicing (**Figure 1**). LncRNAs are divided into positive-sense lncRNAs, antisense lncRNAs, intergenic lncRNAs, intronic lncRNAs, bi-directional lncRNAs and enhancer lncRNAs based on the positional relationship with the corresponding genes (60). Studies identified that lncRNAs can modulate gene expression at distinct levels. LncRNAs are competent to bind to chromatin regulators or chromosome modification complexes directing them to specific genomic site. By doing so, lncRNAs influence the three-dimensional conformation of chromosomes and modify their functional status (61, 62). Additionally, lncRNAs have the capacity to recruit specific protein molecules through direct chromosomal binding, thus modulating the expression of parental genes in subsequent generations (63). LncRNAs can act as miRNA sponges, sequestering them from their intended targets and thereby antagonizing the binding of these miRNAs to their targets (61, 62). Moreover, lncRNAs can also bind to mRNAs, preventing them from associating with other proteins, thereby disrupting the conventional transcription process (64). Furthermore, lncRNAs can interact with proteins to modulate the transcription and post-transcriptional regulation of genes (65, 66).

### 2.3 CircRNAs

CircRNAs are distinct from linear RNAs because they are mainly generated from pre-mRNAs by reverse shear processing (67, 68). Among them, intron sequence pairing, RNA-binding proteins, exon jump cyclization, and off-branching intron lassoing contribute to the loop formation of circRNA (**Figure 1**). Previous studies identified that circRNAs can be produced into three categories: ciRNAs (containing only introns), ElciRNAs (containing both introns and exons), and circRNAs (containing only exons) (69, 70). Nevertheless, there exist additional sources of circRNAs with more specialized origins, such as fusion gene-derived circRNAs, transcriptional read-through circRNAs, and mitochondrial DNA-derived circRNAs. They also play essential roles in altering disease phenotypes or influencing cancer development. The biological functions of circRNAs encompass acting as miRNA sponges to regulate gene transcription, influencing mRNA splicing, interacting with proteins, and potentially undergoing translation (71–73).

## 3 The overview of exosomes

Intracellular multivesicular bodies (MVBs) are released by plasma membrane outgrowth or fusion with membrane vesicles and can form exosomes (74, 75). Exosomes are present in most body fluids and contain large amounts of biologically active substances such as ncRNAs, mRNAs, DNA and proteins and other molecules. They can efficiently mediate inter-cellular and inter-organismal communication through the transport of specific substances including them (76, 77).

### 3.1 The biogenesis and release of exosomes

**Figure 2:**
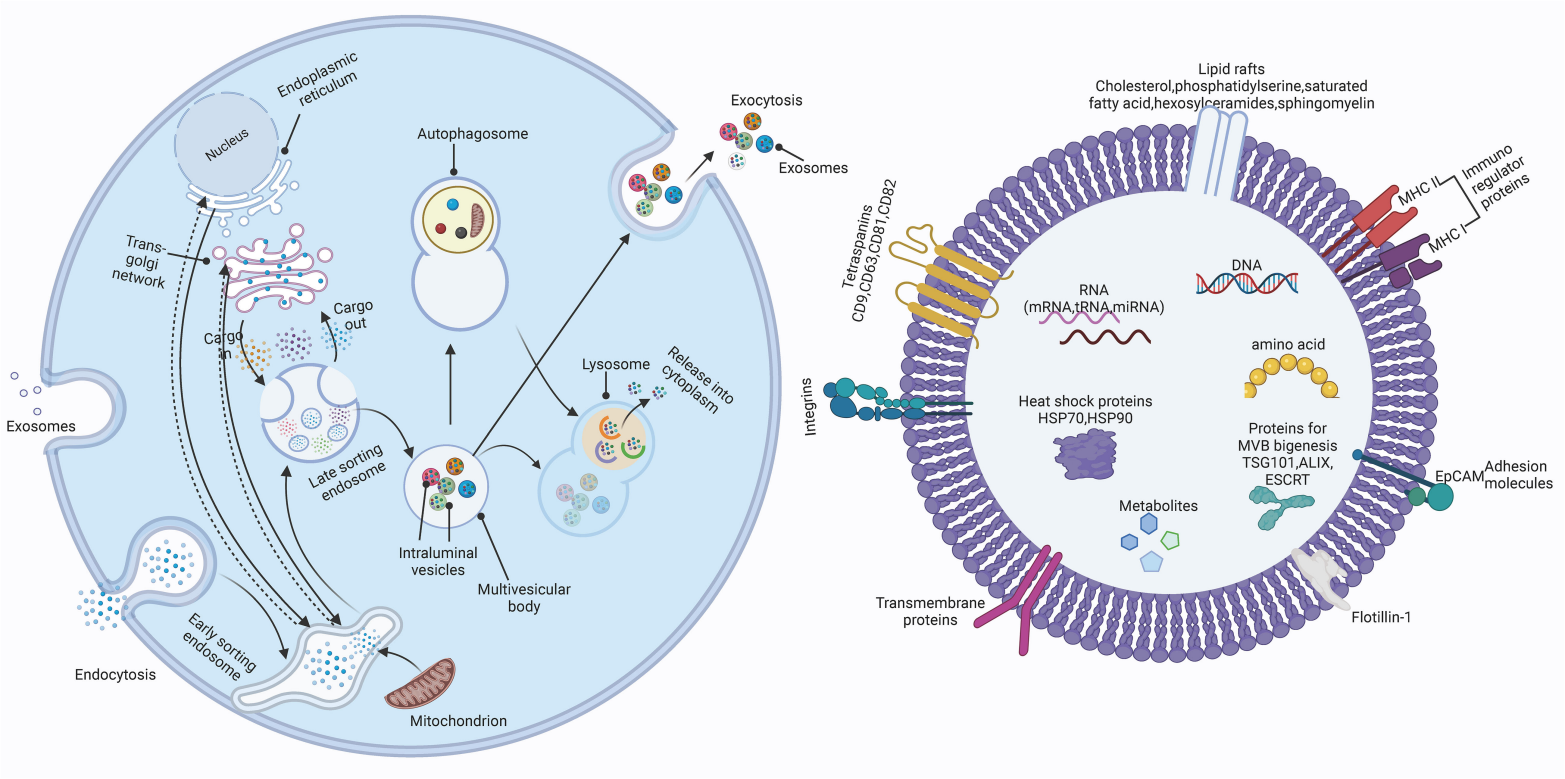
The biogenesis of exosomes. Firstly, the invagination of the cytoplasmic membrane forms a cup-shaped or saucer like structure, which wraps some extracellular components (Specially sorted proteins, nucleic acids, lipids) and membrane proteins together to form an early supporting endosomes (ESEs). ESEs can undergo material exchange with other organelles or fuse with different endosomes to form late supporting endosomes (LSEs). Subsequently, LSEs further form multiple intravascular vesicles (MVBs), which contain many intracavitary vesicles (ILVs). Eventually, MVBs can be degraded by fusing with autophagosomes or lysosomes, or releasing the contained ILVs as exosomes by fusing with the plasma membrane. (The figure was generated by Biorender).

**Figure 3:**
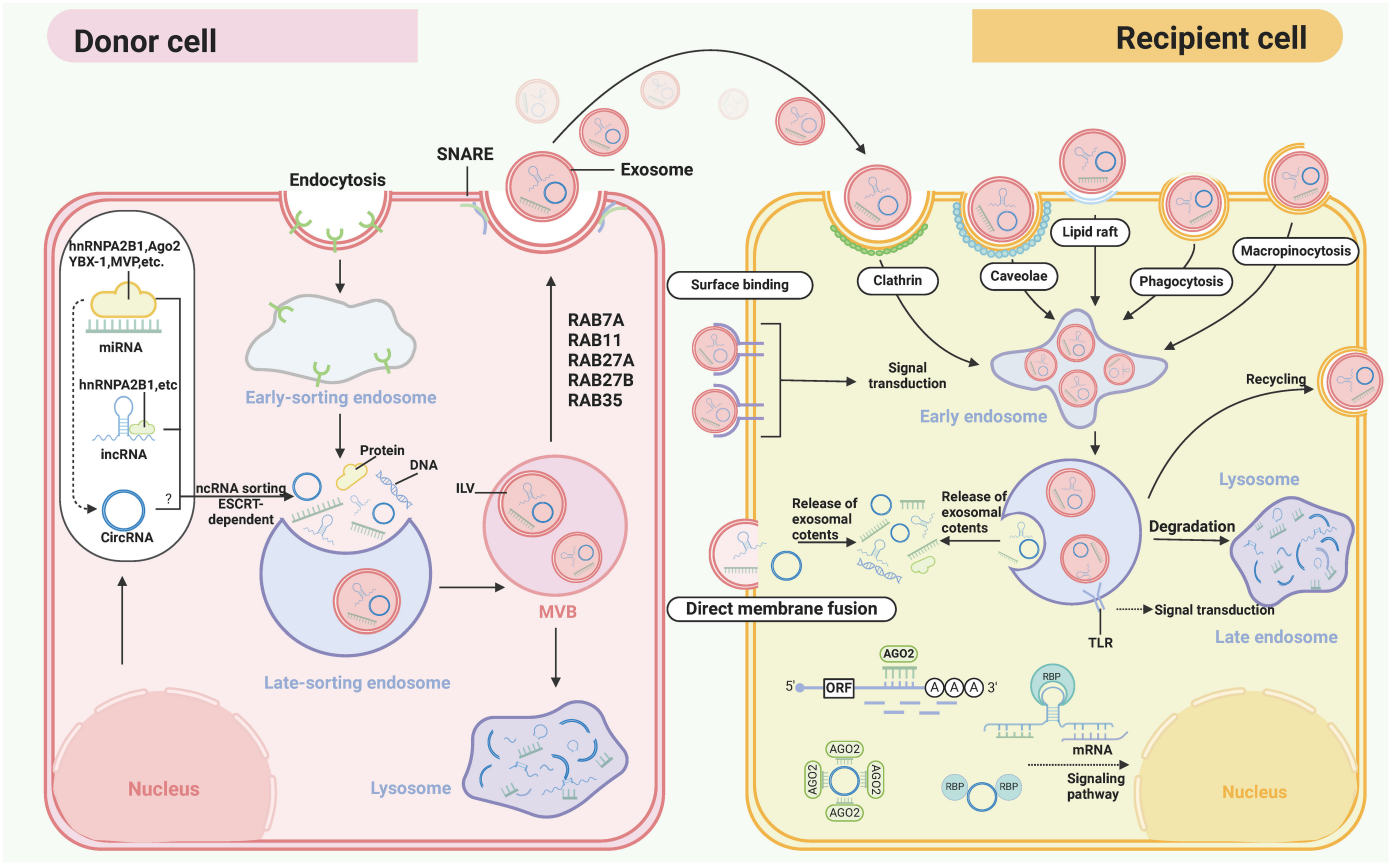
The transmission process of exosomal ncRNAs from the donor cell to the recipient cell. The generation of multivesicular endosomes (MVBs) and intraluminal vesicles (ILVs) include dependent endocytosome sorting complex (ESCRT) mechanism and independent endocytosome sorting complex mechanism. ESCRT is a protein complex located on the cytoplasmic side of endosomes. The main function of ESCRT is to sort specific components and enter ILVs to form precursors for extracellular vesicles. ESCRT-0 recognizes and separates ubiquitinated intracellular membrane transmembrane proteins. The synergistic effect of ESCRT-I and ESCRT-II enables the endocytic membrane to encapsulate specific contents through inward budding. ESCRT III will cut open the formed vesicles. In addition, many RNA binding proteins (RBPs) can bind to specific motifs of miRNAs and lncRNAs (such as GGAG and CCCU) and embed them into extracellular vesicles. Some ncRNAs can be sorted into exosomes mediated by endosomal sorting complex required for transport (ESCRT)-II subcomplex or RBPs recognition to accomplish intercellular delivery through secretion by donor cells and uptake by recipient cells. (The figure was generated by Biorender).

Exosomes are small extracellular vesicles (sEVs) and mainly formed by the secretion of MVBs (78, 79). Initially, the cytoplasmic membrane undergoes invagination, resulting in the formation of a cup-shaped or saucer-like structure, which encloses extracellular components and membrane proteins, thereby giving rise to early sorting endosomes (ESEs). These ESEs can undergo material exchange with other organelles or fuse with different endosomes, eventually leading to the formation of late supporting endosomes (LSEs). Subsequently, LSEs further mature into multivesicular bodies (MVBs), which harbor numerous intraluminal vesicles (ILVs). Ultimately, the majority of MVBs fuse with lysosomes, resulting in the degradation of their contents. However, a minority of MVBs possess CD9, CD63, CD81, among others, facilitating their fusion with the cytoplasmic membrane and releasing extracellular vesicles. The generation of MVBs and ILVs involves two mechanisms: dependent on endosomal sorting complexes required for transport (ESCRT) and independent of ESCRT. ESCRT primarily functions to sort specific components into ILVs, thus serving as precursors for EVs. Conversely, non-endocytic sorting complexes operate independently of ESCRT and facilitate the generation of ILVs and MVBs through the involvement of lipids, ceramides, tetratransmembrane protein families, or heat shock proteins. The biogenesis of exosomes was showed in **Figure 2**. The exosomes are widely distributed in various body fluids such as blood, urine, saliva, and breast milk. They carry a multitude of cellular components, including proteins, DNA, lipids, mRNAs, and ncRNAs (80, 81), which are transferred from donor cells to recipient cells. Exosomes can serve as valuable mediators of intercellular communication in both physiological and pathological states. Besides, several mechanism may modulate the release patterns of exosomes (**Figure 3**). During the biogenesis of exosomal ncRNAs, proteins and short nucleotide sequences interacting with mRNAs in exosomes and heterogeneous nuclear ribonucleoprotein A2/B1 (hnRNPA2/B1) can induce the sorting of ncRNAs into exosomes. HNRNPA2/B1 present in exosomes can straightly bind specifically to miRNAs and lncRNAs through linked specialized motifs (GGAG or CCCU) and monitor their loading into exosomes (82–84). Additionally, circRNAs are roundly distributed in subcellular organelles other than mitochondria and recognize RNA binding proteins (RBPs) that can selectively package circRNAs enriched in purine 5’-GMWGVWGRAG-3’ motifs (85, 86). After packaging, the ncRNAs carried by exosomes from the donor cells can be transferred to distant recipient cells, playing consequential roles in many processes such as growth, progression, immune response, tumor progression, and neurological disorders.

### 3.2 The biological function of exosomes in cancers

Previously, exosomes were considered mere cellular debris produced by cells to eliminate waste. However, mounting evidence has demonstrated that exosomes serve as a valuable mode of long-distance communication between cells within living organisms (79). Exosomes are exert in most of the biological fluids and can transfer functional molecules from parental cells to other cells (87). Exosomes can serve as diagnostic and prognostic biomarkers for various types of tumors. They are enriched with cell-specific proteins, nucleic acids, and lipids, offering a rich molecular source for screening cancer-specific biomarkers (88, 89). In addition, the phospholipid bilayer membrane structure of exosomes can protect their contents from degradation by external proteases and nucleases, resulting in higher stability and specificity compared to serum and urine (90). Moreover, exosomes are widely present in various bodily fluids such as blood, urine, saliva, and milk, and can be obtained through non-invasive means. Furthermore, exosomes can be secreted by tumor cells and delivered into other cells, thus contributing to the growth and metastasis of tumors (91, 92). Exosomes are involved in the regulation of TME and can make tumor cells evade immune surveillance (93, 94). On top of that, they also have the ability to induce neoangiogenesis and can provide nutrient access and support tumor cells in their proliferation (95, 96).

Tumor resistance arises from a multifaceted process involving increased drug efflux, drug inactivation, alterations in drug targets, and inhibition of cell apoptosis. Exosomes play a crucial role in tumor resistance by modulating various mechanisms through the transfer of ncRNAs. Exosomal ncRNAs shield themselves from cytotoxic damage by boosting the efflux of chemotherapy drugs or encapsulating them. Originating from tumors, exosomal ncRNAs exhibit the capability to horizontally spread and combat drug resistance, transmitting it to sensitive cells and fostering the emergence of new drug-resistant cells. Moreover, exosomal ncRNAs evade the harmful effects of cytotoxic drugs through immune evasion, enabling tumors to escape immune surveillance and the cytotoxic effects of drugs. Additionally, exosomal ncRNAs induce chemotherapy resistance in target cells by enhancing autophagy and suppressing apoptosis.

## 4 The roles and mechanism of exosomal ncRNAs in therapeutic resistance of gynecologic tumors

For the moment, the treatment of most kinds of tumors needs a combination of antitumor drugs and local therapy. With the widespread application of antitumor drugs, the improvement of resistance is inevitable. Neglecting the tumor type or treatment regimen chosen, the acquirement of resistance is a highly complicated process. Tumors develop resistance to antitumor drugs through different kinds of mechanisms, and there may be multiple resistance mechanisms in cancers. Studies have demonstrated that tumor drug resistance is associated with cellular mechanisms, like DNA repair, autophagy, metabolic reprogramming, expression of multidrug resistance genes, and epithelial mesenchymal transition (97–99). Studies have shown that exosomal ncRNAs may act as vital roles in the therapeutic resistance including chemoresistance, targeted therapy resistance, radiotherapy resistance and immunotherapy resistance of gynecologic cancers.

### 4.1 Exosomal ncRNAs in gynecological cancers chemotherapy resistance

Chemoresistance is separated into premier and obtained resistance, with chief resistance referring to cancer evasion with initial treatment (100). Clinically, nonresponders are individuals whose tumors fail to shrink after the initiation of therapy. This lack of response is typically attributed to intrinsic resistance caused by preexisting genetic mutations or drug-resistant cellular states within untreated tumors, or the ability of cells to rapidly adapt to therapy (101). Acquired resistance arises when a tumor, which initially responded to therapy, becomes resistant after prolonged treatment. This resistance develops due to mutations in specific tumor cell genes, rendering the tumor insensitive to the anticancer drug. Overcoming chemoresistance poses a significant challenge in the chemotherapy of gynecological cancers. Investigating the potential mechanisms underlying chemoresistance is crucial for improving the prognosis of patients with gynecological cancers (33). Notably, growing evidence demonstrates that exosomal ncRNAs are vital regulators of the chemoresistance of gynecological cancers (**Table 1**). Exosomal ncRNAs derived from OC could transfer drug resistance from resistant cells to sensitive cells, thereby generating new drug-resistant cells. In addition, exosomal ncRNAs could also induce chemotherapy resistance in OC cells by enhancing autophagy and inhibiting apoptosis. Zhao et al. detected the expression patterns of circRNAs in cisplatin (DDP)-sensitive and DDP-resistant OC tissues through microarray technology. They found that the expression of circ-Cdr1as was significantly decreased in both tissues and cell lines of DDP-resistant patients, as well as serum exosomes. Moreover, mechanistic experiments demonstrated that Cdr1as could suppress cell proliferation and sensitize OC cells to DDP-induced apoptosis via regulating the miR-1270/SCAI signaling pathway (102). Lou et al. revealed that circFoxp1 was upregulated in serum exosomes of epithelial ovarian cancer (EOC) patients and positively correlated with the clinical features of EOC. Besides, circFoxp1 could enhance cell proliferation and contribute to the DDP resistance by binding to miR-22 and miR-150-3p and thus elevating CEBPG and FMNL3 expression (103). Wu et al. measured the expression patterns of circRNAs in samples from platinum-resistant and platinum-sensitive OC patients by RNA sequence analysis. They found that hsa_circ_0010467 was markedly upregulated in platinum-resistant tissues, serum exosomes and OC cells. Besides, hsa_circ_0010467 expression was positively correlated with the advanced tumor stage and poor prognosis of OC patients. The results of mechanistic experiments proved that hsa_circ_0010467 could contribute to the maintenance of platinum resistance via acting as a miR-637 sponge to activate the LIF/STAT3 signaling pathway. Moreover, the biogenesis of hsa_circ_0010467 in OC cells could be enhanced by AUF1 (104). Zhuang et al. confirmed that miR-21-5p was upregulated in both DDP-resistant tissues and cell lines. Exosomal miR-21-5p prominently inhibited the DDP sensitivity of SKOV3 cells and facilitate the viability and glycolysis of SKOV3 cells via targeting PDHA (105). Zhang et al. discovered the therapeutic effect of M1 macrophage exosomes containing DDP derived from umbilical cord blood (UCB) on OC and DDP resistance. The mechanism experiment results showed that M1 macrophage exosomes could upregulate PTEN protein and inhibit the expression of miR-130a and Pgp by transmitting H19, thereby reversing DDP resistance (112). The expression level of PANDAR was significantly increased in OC patients with p53 mutations and could be transmitted outward through exosomes. In addition, PANDAR was highly expressed in exosomes of OC cell lines and could promote cell survival and DDP resistance, leading to tumor progression and metastasis. The experimental results of the mechanism showed that SRSF9 could be recruited into nucleosomes by increasing PANDAR and inhibiting cell apoptosis of DDP, and increase the expression of SIRT4/SIRT6 (113).

**Table 1:** Potential roles and mechanism of exosomal ncRNAs in the chemoresistance of gynecological cancers.

Cancers	ncRNAs	Parent cell/source	Target cell	Target	Biological function	Reference
Ovarian cancer	circ-Cdr1as	Serum	/	circ-Cdr1as/miR-1270/SCAI	Inhibit cell proliferation and promote cisplatin induced cell apoptosis	(102)
	circ-Foxp1	Serum	/	circ-Foxp1-miR-22/miR-150-3p-CEBPG/FMNL3	Promote cell proliferation and induce DDP resistance	(103)
	hsa_circ_0010467	Serum	/	AUF1/hsa_circ_0010467/miR-637/LIF/STAT3	Promote cell proliferation and induce DDP resistance	(104)
	miR-21-5p	DDP-resistant cells	DDP-sensitive cells	miR- 21-5p/PDHA	Promote cell vitality, glycolysis and induce DDP resistance	(105)
	lnc-UCA1	Serum	/	lnc-UCA1/miR-143/FOSL2	Promote cell proliferation and inhibit cisplatin	(106)
	miR-98-5p	CAFs	OC cells	miR-98-5p/CDKN1A	Induce DDP resistance	(107)
	miR-548aq-3p	DDP-resistant cells	DDP-sensitive cells	miR-548aq-3p/MED12	Promote cell proliferation and induce DDP resistance	(108)
	miR-6836	DDP-resistant cells	DDP-sensitive cells	TEAD1/miR-6836/DLG2/Yap1	Promote cell stemness and inhibit cisplatin induced cell apoptosis	(109)
	miR-1246	PTX-resistant cells	PTX-sensitive cells	miR-1246/Cav1	Promote cell proliferation and inhibit PTX induced cell apoptosis	(110)
	miR-146a	hUCMSC	OC cells	miR-146a/LAMC2/PI3K/Akt	Inhibit cell proliferation and promote PTX and DTX induced cell apoptosis induced cell apoptosis	(111)
	H19	M1 macrophages	OC cells	H19/miR-130a/Pgp/PTEN	Inhibit DDP resistance	(112)
	PANDAR	p53 mutant OC cells	p53 wild OC cells	SRSF9/PANDAR/SIRT4/SIRT6	Promote cell survival and DDP resistance	(113)
	PLADE	DDP-resistant cells	DDP-sensitive cells	PLADE/HNRNPD/VHL	Promote cell proliferation, migration, invasion and induce apoptosis and DDP resistance	(114)
Cervical cancer	circ_0074269	DDP-resistant cells	DDP-sensitive cells	circ_0074269/miR-485-5p/TUFT1	Promote cell proliferation, migration and induce apoptosis and DDP resistance	(115)
	lnc-HNF1A-AS1	DDP-resistant cells	DDP-sensitive cells	HNF1A-AS1/miR- 34b/TUFT1	Promote cell proliferation and inhibit cisplatin induced cell apoptosis	(116)
	lnc-MALAT1	DDP-resistant cells	DDP-sensitive cells	lnc-MALAT1/miR-370-3p/STAT3/PI3K/Akt	Promote cell proliferation and inhibit cisplatin induced cell apoptosis	(117)
	miR-651	DDP-sensitive cells	DDP-resistant cells	miR-651/ATG3	Inhibit cell proliferation and promote cisplatin induced cell apoptosis	(118)
	miR-320a	/	/	miR-320a/MCL1	Inhibit DDP resistance	(119)
	circSYT15	DDP-resistant cells	DDP-sensitive cells	circSYT1/miR-503-5p/RSF1	Promote cell proliferation, DDP resistance and induce cell apoptosis	(120)

Tumor cells derived exosomal ncRNAs could modulate the evasion of apoptosis, thus contributing to the failure of cancer therapy. Chen et al. identified that the expression of circ_0074269 was prominently decreased in DDP-resistant CC specimen and cells. Inhibition of circ_0074269 in DDP-resistant CC cells surged DDP sensitivity, repressed the proliferation and migration of cells and aroused apoptosis of cells. Mechanistic experiments suggested that circ_0074269 could bind to miR-485-5p to elevate the expression level of TUFT1. In addition, circ_0074269 was enriched in exosomes of DDP-resistant CC cells and could be transmitted into DDP-sensitive CC cells (115). Li et al. identified that UCA1 expression was significantly elevated in OC tissues and serum exosomes of DDP-resistant patients and DDP-resistant cell lines. Suppression of UCA1 could inhibit cell proliferation and promote DDP-induced apoptosis. Mechanistic experiments demonstrated that UCA1 could regulate DDP resistance in OC by modulating the miR-143/FOSL2 pathway (106). Luo et al. showed that HNF1A-AS1 expression was notably elevated in the HeLa/DDP cells, whereas inhibition of HNF1A-AS1 suppressed cell proliferation and enhanced cell apoptosis. Mechanistic experiments showed that exosomal HNF1A-AS1 could promote the expression of TUFT1 by binding to miR-34b. Besides, inhibition of exosomal HNF1A-AS1 in combination with DDP could inhibit tumor increase in nude mice (116). Hu et al. discovered that the lncRNA Metastasis-associated lung adenocarcinoma transcript 1 (MALAT1) expression was elevated in CC tissues, DDP-resistant cells and exosomes derived from CC tissues. Inhibition of MALAT1 notably suppressed cell proliferation and enhanced DDP-induced apoptosis. Mechanistic investigations demonstrated that MALAT1 acted as a sponge for miR-370-3p to increase its expression level of STAT3 and regulate the PI3K/Akt signal pathway, suggesting that MALAT1 could be a promising therapeutic target for treating DDP-resistant CC (117). Guo et al. discovered that CDKN1A expression was higher in DDP-sensitive OC cell lines than that in DDP-resistant cells. Silencing of CDKN1A in OC cells facilitated cell proliferation, cycling and reduced apoptosis. Mechanistic experiments implied that miR-98-5p was increased in CAF-derived exosomes and could promote DDP resistance in OC via targeting CDKN1A (107). Zhu et al. verified that miR-651 expression was markedly lower in the circulating peripheral blood of CC patients compared to healthy controls with a beneficial diagnostic efficacy (AUC = 0:9050). Besides, miR-651 was down-regulated in HeLa/DDP cells and inhibition of miR-651 could suppress DDP resistance, proliferation and promote apoptosis in HeLa cells. Moreover, HeLa/DDP cells could transferred exosomes into HeLa/S cells and contribute to the malignancy of HeLa/S cells. Overall, cancer-derived exosomal miR-651 could target ATG3 to block DDP resistance (118). Zhao et al. investigated that miR-484 expression was decreased in both OC cells and angiogenic endothelial cells. Incubation of cells with miR-484-containing RGD-modified exosomes promoted sensitization of OC cells to chemotherapy-induced apoptosis through vascular normalization. Mechanistic experiments implied that miR-484 could decrease the expression of VEGFA and the corresponding receptor in endothelial and cancer cells, thus prolonging the survival time of tumor-bearing mice after chemotherapy (121). Zhou et al. proved that the expression level of miR-320a was decreased in both DDP-resistant CC tissues and cell lines. Treatment with engineered miR-320a exosomes could attenuate DDP resistance via targeting MCL1 in CC cells (119). Wang et al. detected the exosomal miRNA expression profiles in OC and identified that exosomal hsa-miR-675-3p acted as a latent target for repressing DDP resistance of OC (122). Shi et al. confirmed that MED12 expression was markedly decreased in DDP-resistant OC cells. Co-culture of DDP-resistant cells with parental OC cells declined the expression of MED12 in the cells as well as the sensitivity to DDP. Besides, miR-548a-3p was upregulated in the exosomes of DDP-resistant OC cells and promoted the survival and proliferation rate of OC cells under cisplatin treatment via targeting MED12 (108). Zou et al. identified that miR-6836 expression was increased in EOC positively correlated with poor chemotherapeutic response and low survival rates of EOC patients. Overexpression of miR-6836 could boost the DDP-resistance of EOC cells by rising stemness and inhibiting apoptosis. Mechanistic experiments suggested that miR-6836 was modulated by TEAD1 and can target DLG2 to sharpen up the nuclear translocation of Yap1. Additionally, miR-6836 could be encapsulated into the exosomes of DDP-resistant EOC cells and transmitted to DDP-sensitive EOC cells to reverse their cisplatin responses (109). The expression of PLADE was significantly decreased in high grade serous OC (HGSOC) swapped ascites exosomes, tumor tissues, and corresponding metastatic tumors in DDP resistant patients. HGSOC patients with high levels of PLADE expression exhibited longer progression free survival. The *in vitro* experimental results showed that PLAD could promote DDP sensitivity by inhibiting cell proliferation, migration, and invasion, as well as enhancing cell apoptosis. Mechanistically, PLADE could bind to heteronuclear ribonucleoprotein D (HNRNPD) through VHL mediated ubiquitination and downregulate to induce an increase in RNA: DNA hybrid (R-loop) quantity and DNA damage (114). Chen et al. confirmed that the expression of circSYT15 was upregulated in DDP resistant CC cells and exosomes. Knocking down circYT15 in CC cells could inhibit cell proliferation, drug resistance and induce CC cell apoptosis. Moreover, exosomal circSYT15 could be transmitted from DDP resistant CC cells to DDP sensitive CC cells and enhance the DDP resistance of CC by targeting the miR-503-5p/RSF1 axis (120).

Exosomal ncRNAs also exerted essential roles in paclitaxel (PTX) resistance in gynecological cancers. Huang et al. revealed that circ_0025033 expression level was elevated in OC tissues and cells, especially in PTX-resistant OC cells. Inhibition of circ_0025033 in OC cells reduced PTX resistance, cell migration and invasion and promoted cell apoptosis. Mechanistic experiments demonstrated that circ_0025033 could upregulate FOXM1 expression via sponging miR-532-3p, which inversely benefited malignant activity in PTX-resistant OC cells (123). Kanlikilicer et al. confirmed that miR-1246 was enriched in exosomes of OC cells and could inhibit cell proliferation through PDGFβ receptors in recipient cells. Besides, the OC patients with high expression of miR-1246 had a markedly worse overall prognosis than that with low expression. It turned out that the expression of miR-1246 in exosomes of PTX-resistant OC was prominently higher than that in PTX-sensitive-OC. Overexpression of Cav1 and anti-miR-1246 could promote the PTX sensitivity of OC cells. Besides, miR-1246 could also be transferred from OC cells to M2-type macrophages (110). Qiu et al. isolated exosomes from hUCMSCs and took advantages of them to treat docetaxel (DTX)-resistant SKOV3 cells and PTX-resistant A2780 cells. The findings of experiments indicated that miR-146a was enriched in hUCMSC-derived exosomes and could inhibit OC cell growth and chemotherapy resistance through targeting the LAMC2/PI3K/Akt signaling pathway (111). Li et al. discovered that exosomal miR-429 expression was elevated in multidrug-resistant SKOV3 cells. SKOV3-derived exosomal miR-429 could be delivered into the A2780 and strengthen the proliferation and drug resistance of cells by modulating calcium-sensing receptor (CASR)/STAT3 pathway (124).

### 4.2 Exosomal ncRNAs in gynecological cancers targeted therapy resistance

Targeted therapy can effectively inhibit the progression of cancers andhas showed excellent therapeutic impacts when used in combination with chemotherapy (125, 126). Malignant tumors can be gradually transformed into chronic diseases, with further deterioration being controlled by targeted drugs. Exosomal ncRNAs were also invplved in the targeted therapy resistance of gynecological cancers. Zhu et al. identified that hypoxic EOC cells could induce macrophages into the tumor-associated macrophages (TAMs)-like phenotype. Macrophages could transfer miR-223-rich exosomes into EOC cells under hypoxia condition. While exosomal miR-233 could enhance chemoresistance through targeting the PTEN-PI3K/AKT signal pathway. Moreover, patients with high exosomal miR-223 levels were positively related to the recurrence of EOC (127). Zhou et al. discovered that exosomes secreted by cervical squamous cell carcinoma (CSCC) cells could deliver miR-142-5p into lymphatic endothelial cells and induce indoleamine 2,3-dioxygenase (IDO) expression via ARID2-DNMT1-IFN-γ signaling. Besides, exosomal miR-142-5p could also inhibit and deplete CD8^+^ T cells (128). Zhou et al. found that miR-765 was down-regulated in EC and decreased by estrogen. Besides, CD45RO-CD8^+^ T cell-derived exosomes could release more miR-765 than CD45RO^+^CD8^+^ T cells, thus repressing the malignant progression of estrogen-driven tumors via modulating the miR-765/PLP2 signal axis (129). MiR-92b-3p expression was obviously decreased in OC cell-derived exosomes compared to ovarian epithelial cells. Besides, peptide-engineered exosomes overexpressing miR-92b-3p had stronger anti-angiogenic and anti-tumor capacities either alone or in combination with apatinib in OC (130).

### 4.3 Exosomal ncRNAs in gynecological cancers radiotherapy resistance

Radiotherapy is one of the most commonly used therapeutic approaches for gynecological cancers. However, radiotherapy resistance that arises during the treatment process remains a challenging problem (131, 132). Studies have verified that exosomal ncRNAs participate in the radiotherapy resistance of gynecological cancers. Gu et al. revealed that M2-polarized macrophages were able to transmit exosomal hsa_circ_0001610 into EC cells to significantly repress the radiosensitivity of OC cells. Mechanistic experiments showed that hsa_circ_0001610 could elevate the expression of cell cycle protein B1 by binding to miR-139-5p (133). Konishi et al. identified that co-culture of SKG-II and C4-I cells with exosomal miR-22 could promote the radiosensitivity of CC cells by targeting c-Myc binding protein (MYCBP) and human telomerase reverse transcriptase (hTERT) (134). MiR-1323 expression was increased in CC cells and also enriched in the exosomes of CAFs. CAFs could transfer miR-1323 into CC cells and facilitate the proliferation, migration, invasion, and radiosensitivity of CC cells by targeting poly(A)-binding protein nucleus 1 (PABPN1)/IGF2BP1/glycogen synthase kinase 3 beta (GSK-3β)/Wnt/β-catenin signal pathway (135).

### 4.4 Exosomal ncRNAs in gynecological cancers immunotherapy resistance

Immunotherapy have achieved great clinical responses in recent years. Immune infiltrating cells in the TME exert crucial roles in tumor occurrence and development. Exosomal ncRNAs were also associated with the immunotherapy resistance of gynecological cancers. Zhou et al. confirmed that miR-1468-5p was enriched in exosomes of CC cells and could be delivered into cancer-associated lymphatic endothelial cells. Besides, exosomal miR-1468-5p could impaire T-cell immunity by promoting lymphoid PD-L1 upregulation and lymphangiogenesis and modulating JAK2/STAT3/homology-containing box 1 (HMBOX1)/SOCS1 pathway (136). MiR-29a-3p was enriched in EVs of TAMs and could be delivered into OC cells. Mechanistic experiments demonstrated TAM-derived exosomal miR-29a-3p could facilitate OC cell proliferation and immune escape by modulating the FOXO3-AKT/GSK3b pathway and promoting PD-L1 expression (137). Gao et al. found that miR-124 could directly target MCT1 and reduce lactate uptake, thereby weakening the immunosuppressive ability of Treg cells in OC. In addition, exosomal miR-124 derived from bone marrow mesenchymal stem cells (BM-MSCs) could repress tumor growth and promote response to PD-1 blockade therapy (138).

## 5 Exosomal ncRNAs as promising diagnostic and prognostic biomarkers

With the advancement of liquid biopsy methods and extensive research, exosomes are regarded as significant biomarkers in the diagnosis and prognosis of various of diseases (139, 140). Exploration implied that exosomal ncRNAs could also act as promising diagnostic and prognostic biomarkers in gynecological cancers (**Tables 2**, **3**).

**Table 2:** Potential of exosomal ncRNAs as diagnostic and prognostic tools in ovarian cancer.

Exosomal ncRNAs	Sample size (Normal : Tumor)	Detection Method	p value	Diagnosis	FIGO stage (p value)	Histological grade (p value)	LNM (p value)	DM (p value)	Tumor position (p value)	OS (p value)	DFS (p value)	Follow-up (months)	Reference
circ-0001068	(53: 95)	Specific qRT-PCR	p< 0.001	AUC=0.9697	/	/	/	/	/	/	/	/	(141)
MALAT1	(60: 60)	Specific qRT-PCR	p< 0.001	/	p< 0.001	p=0.035	p< 0.002	/	/	p< 0.05	/	80	(142)
miR-1290	(13: 70)	Specific qRT-PCR	p> 0.05	AUC=0.48	/	/	/	/	/	/	/	/	(143)
miR-1307	(50: 50)	Specific qRT-PCR	p< 0.05	AUC=0.694	p=0.0455	/	p=0.9000	p=0.9579	/	/	/	/	(144)
miR-375			p< 0.05	AUC=0.788	p=0.5625	/	p=0.0376	p=0.4725	/	/	/	/	(144)
miR-145	(20: 48)	Specific qRT-PCR	p< 0.001	AUC=0.910	p=0.524	/	p=0.477	p=0.042	/	/	/	/	(145)
miR-200c			p< 0.001	AUC=0.802	p=0.951	/	p=0.708	p=0.077	/	/	/	/	
miR-21			p< 0.001	AUC=0.585	p=0.079	/	p=0.843	p=0.033	/	/	/	/	
miR-93			p=0.303	AUC=0.755	p=0.127	/	p=0.016	p=0.748	/	/	/	/	
miR-484	(60: 113)	Specific qRT-PCR	p< 0.05	AUC=0.821	p< 0.001	p=0.0035	p=0.0012	/	/	/	/	/	(146)
miR-34a	/	Specific qRT-PCR	p< 0.05	/	/	/	p< 0.05	/	/	/	/	/	(147)
miR-4732-5p	(21: 34)	Specific qRT-PCR	p = 0.018	AUC=0.889	/	/	/	/	/	/	/	/	(148)
miRNA-205	(31: 36)	Specific qRT-PCR	p< 0.05	AUC=0.715	p=0.032	/	p=0.007	/	/	/	/	/	(149)
miRNA-1290	(15: 67)	Specific qRT-PCR	p< 0.05	AUC=0.749	/	/	/	/	/	/	/	/	(150)
miR-1260a	(130: 132)	Specific qRT-PCR	p< 0.05	AUC=0.7551	p=0.8807	/	p=0.2278	p=0.6135	p=0.4045	/	/	/	(151)
miR-7977			p< 0.05	AUC=0.6982	p=0.6080	/	p=0.1962	p=0.7022	p=0.4045	/	/	/	
miR-192-5p			p< 0.05	AUC=0.8264	p=0.4718	/	p=0.3609	p=0.6186	p=0.5912	/	/	/	
miR-320d	(173: 166)	Specific qRT-PCR	p< 0.05	AUC=0.6549	p=0.0179	/	p=0.0008	p=0.0887	p=0.3948	/	/	/	(152)
miR-4479			p< 0.05	AUC=0.7781	p=0.0301	/	p=0.0132	p=0.5507	p=0.4604	/	/	/	
miR-6763-5p			p< 0.05	AUC=0.6834	p=0.1812	/	p=0.0127	p=0.8397	p=0.7399	/	/	/	

**Table 3:** Potential of exosomal ncRNAs as dianostic and prognostic tools in cervical cancer and endometrial cancer.

Exosomal ncRNAs	Sample size (Normal : Tumor)	Detection Method	p value	Diagnosis	FIGO stage (p value)	Differentiation (p value)	LNM (p value)	DM (p value)	HPV infection (p value)	OS (p value)	RFS (p value)	Follow-up (months)	Reference
DLX6-AS	(110: 114)	Specific qRT-PCR	p< 0.001	AUC=0.892	p< 0.001	p=0.0004	p=0.0071	/	p=0.0657	p=0.0076	p=0.002	60	(154)
let-7d-3p	/	droplet digital PCR	p< 0.05	AUC=0.822	/	/	/	/	/	/	/	/	(49)
miR-30d-5p			p< 0.05	AUC=0.79	/	/	/	/	/	/	/	/	
let-7d-3p	(87: 97)	Specific qRT-PCR	p< 0.05	AUC=0.770	/	/	/	/	/	/	/	/	(2)
miR-30d-5p			p< 0.05	AUC=0.752	/	/	/	/	/	/	/	/	
let-7d-3p			p< 0.05	AUC=0.721	/	/	/	/	/	/	/	/	
miR-30d-5p			p< 0.05	AUC=0.677	/	/	/	/	/	/	/	/	
miR-125a-5p	(28: 44)	Specific qRT-PCR	p< 0.001	AUC=0.7129	p=0.0271	/	p=0.1978	/	p=0.0418	/	/	/	(155)
miR-15a-5p	(87: 115)	droplet digital PCR	p< 0.001	AUC=0.823	/	/	/	/	/	/	/	/	(162)
miR-106b-5p			p=0.0055	AUC=0.611	/	/	/	/	/	/	/	/	
miR-107			p< 0.001	AUC=0.682	/	/	/	/	/	/	/	/	

### 5.1 OC

Wang et al. utilized microarray technology to detect differential expression of circRNAs in exosomes from the serum of OC patients and healthy controls. Their findings revealed significant upregulation of circ-0001068 in serum exosomes of OC patients compared to healthy volunteers. Furthermore, circ-0001068 could be encapsulated into exosomes and transferred to T cells, and within T cells, it could promote PD1 expression by binding to miR-28-5p contributing to tumor immune evasion. Overall, circ-0001068 in OC exosomes could have clinical implications. Exosomal circ-0001068 could serve as a novel biomarker for OC diagnosis and monitoring, and as a therapeutic target to enhance immune responses against OC (141). Qiu et al. discovered that the expression of MALAT1 was elevated in the exosomes derived from metastatic EOC cells. Besides, exosomal MALAT1 could be transferred into human umbilical vein endothelial cells (HUVECs). Moreover, the expression of exosomal MALAT1 were obviously associated with advanced and metastatic phenotypes of EOC. The prognostic nomogram model prohibited a good prediction of the probability of 3-year OS in patients with EOC based on the c-index and calibration curve of exosomal MALAT1 (142). Li et al. detected the expression patterns of lncRNAs and mRNAs in exosomes of OC patients’ serum. The results indicated that totally 117 lncRNAs and 513 mRNAs were differentially expressed. Moreover, the results of bioinformatics analysis demonstrated that exosomal DIO3OS could regulate OC progression and disease survival by modulating hsa-miR-27a-3p-HOXA10 signal axis (153). Kobayashi et al. identified that miR-1290 expression was markedly increased in exosomes of high-grade plasmacytoid OC (HGSOC) patients’ serum. Moreover, the expression level of exosomal miR-1290 could effectually discriminate HGSOC from other histological kinds of malignant tumors (AUC=0.76; specificity: 85%; sensitivity: 47%). 85%; sensitivity: 47%) and normal controls (AUC=0.71; specificity: 85%; sensitivity: 63%) (143). Su et al. verified that both miR-1307 and miR-375 were notably upregulated in exosomes of OC patients’ serum. Either exosomal miR-1307 or exosomal miR-375 had good diagnostic efficacy by AUC (AUC 0.694 and 0.788, respectively), and the AUC for the combination of the couple was 0.837. Moreover, the expression level of exosomal miR-1307 was positively related to the tumor stage, while exosomal miR-375 was associated with lymph node metastasis (LNM) (144). Kim et al. verified that the expression of miR-93, miR-145 and miR-200c in serum exosomes were obviously higher in OC than that of negative control. The AUCs of cancer antigen 125 (CA125), miR-145, miR-200c, miR-21, and miR-93 were 0.80, 0.910, 0.80, 0.585, and 0.755, respectively. Besides, miR-145 was more sensitive (91.6%) and miR-200c was more specific (90.0%) compared to CA125 (145). Zhang et al. confirmed that the expression of miR-484 in exosomes of OC patients’ serum was remarkably decreased. The ROC analysis showed that the AUC of exosomal miR-484 was 0.821 whereas a combined diagnosis of exosomal miR-484 with CA-125 revealed an AUC of 0.912. Besides, the expression of exosomal miR-484 was negatively relevant to the aggressiveness of OC, as well as with OS and PFS (146). The expression of exosomal miR-34a levels were significantly elevated in early OC patients’ serum compared with those with progressed OC, and negatively correlated to the LNM and recurrence of OC (147). Liu et al. (148) identified that plasma derived exosomal miR-4732-5p could be applied as a possible biomarker for monitoring the boom of EOC (AUC = 0.889; sensitivity = 85.7% and specificity = 82.4%, p= 0.018) (148). Zhu et al. confirmed that miR-205 was upregulated in exosomes of OC patients’ plasma, especially in stage III-IV with lymph node metastasis. Exosoaml miR-205 could serve as a significant biomarker (AUC = 0.715; sensitivity: 66.7% and specificity: 78.1%) for OC (149). MiRNA-1290 was higher in EOC patients compared to those with benign ovarian tumors. Besides, serum derived exosomal miR-1290 had beneficial diagnostic efficacy with an AUC value of 0.794, sensitivity and specificity of 69.2% and 87.3%, respectively (150). MiR-1260a, miR-7977 and miR-192-5p were obviously decreased in exosomes of EOC patients’ plasma. The diagnostic efficacy of the combination of the three exosomal miRNAs was good and with an AUC of 0.8337 (sensitivity: 70.5% and specificity: 89.1%). Moreover, the expression level of exosomal miR-7977 was decreased in EOC patients with high neutrophil-lymphocyte ratio and correlates with this ratio (151). Wang et al. found that a total of 95 miRNAs especially miR-320d, miR-4479, and miR-6763-5p were differentially expressed in the exosomes from EOC patients’ plasma and healthy controls. ROC analysis demonstrated that these three miRNAs could serve as good diagnostic markers for EOC, with respective AUCs of 0.6549, 0.7781, and 0.6834. Additionally, the levels of these three exosomal miRNAs were significantly related to LNM, while the expression of exosomal miR-320d and miR-4479 correlates with tumor stage (152).

### 5.2 CC

Ding et al. identified that the expression of exosomal DLX6-AS1 was prominently higher in CC patients’ serum and could effectively distinct it from cervical intraepithelial neoplasia (CIN) patients (AUC=0.831) and common controls (AUC=0.892). Besides, the expression of exosomal DLX6-AS1 was positively related to LNM, differentiation, International Federation of Gynecology and Obstetrics (FIGO) stage and shortened survival and tumor recurrence in CC patients (154). Zheng et al. demonstrated that 121 exosomal miRNAs were differentially expressed in healthy individuals, CC patients and CIN patients via using HTS. Moreover, combined diagnosis of exosomal let-7d-3p and exosomal miR-30d-5p could distinguish individuals in the CIN II+ group from those in the CIN I- group (AUC=0.828). Further integration of both miRNAs into a cytology-based assay model yielded an even higher AUC worth of 0.887 (49). Lv et al. found that exosomal miR-125a-5p was downregulated in CC patients’ plasma and with a AUC of 0.7129 (155). Cho et al. identified that miR-1228-5p, miR-33a-5p, miR-3200-3p, and miR-6815-5p expression levels were increased in patients with endometrial polyps and correlated with the extent of metastasis, while miR-146a-3p was decreased (156). Cho et al. confirmed that miR-142-3p and the combination of 5 mRNAs and 8 snoRNAs could markedly differentiate normal samples from cancer group samples (157). The expression levels of miR-374a-5p and miR-431-5p were inversely correlated with tumor-infiltrating CD8^+^ and FOXP3^+^ T cells. Besides, a total of 9 differentially expressed miRNAs could significantly distinct from the recurrence-free and recurrence groups (158).

### 5.3 EC

Exosomes are widely available in various of bodily fluids and are sources of ideal biomarkers for liquid biopsies in many diseases including EC (159–161). Zhou et al, detected the expression levels of exosomal miRNAs in EC patients’ plasma by high-throughput sequencing. They found that miR-15a-5p, miR-106b-5p, and miR-107 was upregulated in exosomes isolated from plasma samples of EC patients. Notably, exosomal miR-15a-5p exhibited a strong correlation with the depth of muscle infiltration, invasiveness of EC, and reproductive hormone levels. It showed an AUC of 0.813 alone and 0.899 in combination with CEA and CA125 (162).

## 6 Discussion and prospects

Gynecological cancers mainly include CC, OC and EC and seriously threaten to the wellness of female, especially to younger populations. Exosomes are heterogeneous nanoscale phospholipid vesicles formed by cells via the mechanism of endocytosis-fusion-exocytosis, secreted by diverse cells (163, 164). Emerging evidence has demonstrated that exosomes are a valued means of long-distance communication between cells in organisms (165, 166), and exert vital roles in various of physiopathologic processes (167, 168). Moreover, exosomes are involved in tumorigenesis, invasion, and metastasis by transporting bioactive substances and serving as information exchange carriers between the TME and tumor cells (169, 170). The bioactive molecules that have been identified in exosomes include proteins, nucleic acids and lipids (171). Exosomes have also emerged as versatile tools in biomarker identification, vaccine development, and targeted drug delivery. Optimization and rational modification of exosomes for therapeutic interventions is underway (172). Aberrant expression of exosome contents can reflect the pathological state of the organism. Exosomes have been reported to be involved in the therapeutic resistance of distinct cancers. Cancer-associated fibroblasts (CAFs) can promote chemoresistance in pancreatic ductal adenocarcinoma cells following gemcitabine treatment by secreting exosomal miR-3173-5p into cancer cells and modulate the signaling communication (173). Adipose-derived exosomes can promote chemoresistance to oxaliplatin via transpotting exosomal microsomal triglyceride transfer protein (MTTP) into cancer cells and thus regulating PRAP1/ZNFE1/GPX4 signal pathway (174). Besides, many investigations have indicated that exosomes and exosome contents can be applied as novel cancer biomarkers (175).

In recent years, numerous exosomal ncRNAs involved in tumorigenesis have been discovered and identified. Exosomal ncRNAs are conserved and stable with greater target specificity, and have the potential to monitor disease progression or recurrence (176–178). Exosomal ncRNAs can also act as therapeutic targets to inhibit tumor metastasis, modulate anti-tumor immune responses and mediate tumor drug resistance (179, 180). But since the research of exosomal ncRNAs is still in its infancy, we still have lots of gaps in diverse aspects of exosomal ncRNAs. Primarily, the specific sorting mechanism of specific ncRNAs into exosomes remains unclear, whether the secretion of exosomal ncRNAs is active transport or passive diffusion, and what molecules may be involved in the regulation. Secondly, the degradation mechanism of exosomal ncRNAs in the organism still needs to be further explored. However, detecting low abundance ncRNAs remains challenging, and there is still a need for more sensitive and efficient methods for exosome extraction. Larger cohort studies are required to confirm the clinical applicability of exosomal ncRNAs as biomarkers.

Therapy resistance in tumor cells is influenced by multiple factors (181, 182), and exosomes have been identified as mediators in the transmission of therapy resistance between cells (22, 183, 184). For instances, hsa_circ_0004085 is upregulated in colon cancer and fusobacterium nucleatum infection can promote hsa_circ_0004085 formation by hnRNP L and packaged hsa_circ_0004085 into exosomes by hnRNPA1. Exosomes secreted by Fn-infected colon cancer cells can deliver hsa_circ_0004085 into recipient cells and delivering resistance to oxaliplatin and 5-FU by modulating GRP78 and ATF6p50 (185). Exosomal miR-146a-5p expression is elevated in urothelial bladder cancer and it can be delivered from CAFs to bladder cancer cells. CAF-derived miR-146a-5p can promote stemness and enhance chemoresistance in urothelial bladder cancer via regulating YY1/SVEP1 and ARID1A/PRKAA2 signal pathways (186). Besides, tumor cell drug resistance and thus chemotherapy efficacy can be declined and thus improved by inhibiting the release of exosomes or altering their active components during the investigation of drug resistance therapy. However, there are still many opportunities and challenges in the application of exosomal ncRNAs in the therapy resistance in gynecologic cancers. For examples, the research on exosomal ncRNAs and tumor drug resistance mainly stays at the *in vitro* level, and there is no linked research report on clinical cases so far. Translating the experimental consequences at the cellular level into clinical trials is still the most difficult challenge. The potential of utilizing exosomes for therapy lies in controlling extracellular information exchange and targeted drug delivery. Further investigations are needed to uncover the specific mechanisms underlying the secretion and delivery of exosomal ncRNAs. On the other hand, it is needful to boost the delivery efficiency of exosomes. With the in-depth research on the mechanism of exosomal ncRNAs regulating therapy resistance in diverse tumors, we hope that in the future we can make satisfactory progress in the areas of tumor prognosis judgment, tumor treatment monitoring and targeted therapy.

## 7 Conclusion

Overall, we reviewed the biological functions and potential molecular mechanisms of exosomal ncRNAs in the therapeutic resistance of gynecologic cancers. discussing Besides, we also identified the potential diagnostic and prognostic value of exosomal ncRNAs in gynecologic cancers.

## Author contributions

CX: Conceptualization, Visualization, Writing – original draft. PX: Data curation, Investigation, Methodology, Validation, Writing – original draft. JZ: Data curation, Investigation, Validation, Writing – original draft. SH: Formal analysis, Investigation, Software, Visualization, Writing – review & editing. TH: Data curation, Investigation, Methodology, Project administration, Validation, Visualization, Writing – review & editing. AH: Conceptualization, Funding acquisition, Writing – review & editing.
